# Maternal Plasma Metabolomic Profiles in Spontaneous Preterm Birth: Preliminary Results

**DOI:** 10.1155/2018/9362820

**Published:** 2018-02-15

**Authors:** Barbara Lizewska, Joanna Teul, Pawel Kuc, Adam Lemancewicz, Karol Charkiewicz, Joanna Goscik, Marian Kacerovsky, Ramkumar Menon, Wojciech Miltyk, Piotr Laudanski

**Affiliations:** ^1^Department of Perinatology, Medical University of Bialystok, Bialystok, Poland; ^2^Department of Pharmaceutical Analysis, Medical University of Bialystok, Bialystok, Poland; ^3^Faculty of Computer Science, Bialystok University of Technology, Bialystok, Poland; ^4^Department of Obstetrics and Gynecology, Faculty of Medicine Hradec Kralove, Charles University in Prague, Hradec Kralove, Czech Republic; ^5^Biomedical Research Center, University Hospital Hradec Kralove, Hradec Kralove, Czech Republic; ^6^Department of Obstetrics & Gynecology and Department of Microbiology, The University of Texas Medical Branch, Galveston, TX, USA

## Abstract

**Objective:**

To profile maternal plasma metabolome in spontaneous preterm birth.

**Method:**

In this retrospective case-control study, we have examined plasma of patient with preterm birth (between 22 and 36 weeks of pregnancy (*n* = 57)), with threatened preterm labor (between 23 and 36 weeks of pregnancy (*n* = 49)), and with term delivery (*n* = 25). Plasma samples were analysed using liquid chromatography quadrupole time-of-flight mass spectrometry (LC-Q-TOF-MS) in positive and negative polarity modes.

**Results:**

We found 168 differentially expressed metabolites that were significantly distinct between study groups. We determined 51 metabolites using publicly available databases that could be subdivided into one of the five groups: amino acids, fatty acids, lipids, hormones, and bile acids. PLS-DA models, verified by SVM classification accuracy, differentiated preterm birth and term delivery groups.

**Conclusions:**

Maternal plasma metabolites are different between term and preterm parturitions. Part of them may be related with preterm labor, while others may be affected by gestational age or the beginning of labor. Metabolite profile can classify preterm or term delivery groups raising the potential of metabolome as a biomarker to identify high-risk pregnancies. Metabolomic studies are also a tool to detect individual compounds that may be further tested in targeted researches.

## 1. Introduction

Preterm birth (PTB) is defined as labor between 22 and 37 weeks of gestation [1]. PTB is one of the biggest challenges of obstetrics, because there is no effective method for screening, early diagnosis for high-risk status, treatment, and prevention [2, 3]. Children born preterm show a high rate of maternal mortality, inflammation, and complications in later life such as neurological, respiratory, gastrointestinal, or hematologic problems [4]. This causes serious consequences, both social and economic [5]. The causality of PTB is unclear and often complex, and therefore screening women for high-risk status has not been successfully developed [6]. Potential causes of PTB include infection, low socioeconomic status, high or low BMI, and prior history of preterm delivery [7–11]. However, in many patients, it comes to PTB and it is not possible to definite risk factors for its occurrence. It is crucial to find a biomarker that will enable to identify patients at risk for PTB.

Various studies have focused on the inflammatory markers associated with PTB [12, 13]. However, it is difficult to identify a novel and universal marker in a targeted research, as such approaches are often biased.

Metabolomic is the method measuring small molecule components—metabolites—taking place in all chemical processes in the organism. These chemical processes might be changed by different stimuli, for example, drug or disease, and the changes are reflected by metabolite concentration in the searched samples [14]. The collection of all metabolites in particular tissue or biofluid is called metabolome.

Every tissue and biological fluid, such as urine, amniotic fluid, or plasma, has characteristic for itself set of metabolites [15].

The most commonly used techniques in metabolomics are H^1^ NMR (proton nuclear magnetic resonance) and chromatographic methods (gas or liquid chromatography) hyphenated with mass spectrometry (GC-MS, LC-MS). On the basis of metabolomic analysis, it is possible to find a single marker discriminating a particular group of patients as well as create a metabolomic profile characterizing the group. Therefore, it is possible to identify metabolic pathways that may be impaired in various disease states, including PTB.

Metabolomic studies looking for markers of various pathologies of pregnancy, including PTB, are becoming increasingly popular. The most frequently researched biological fluid in metabolomic studies, in term delivery (TD) or PTB, is amniotic fluid [16, 17]. The studies comparing metabolites from different biological fluids and performed with different analytical platforms (H^1^ NMR or MS) could give interesting results [16].

Only one study has been conducted on metabolomic changes in the blood of PTB patients by using ultraperformance liquid chromatography-mass spectrometry (UPLC-MS) [18], but in contrary to our research, this study was carried out in serum, not in plasma. However, due to heterogeneity in inclusion criteria that included pregnancies ended with several adverse outcome during pregnancy (preterm birth of small for gestational age neonates (*n* = 3), preterm birth of normal birth weight neonates (*n* = 8), term birth of small for gestational age neonates (*n* = 28), and neonate, who unexpectedly was admitted to neonatal intensive care unit (*n* = 1)), data were not conclusive of spontaneous preterm birth [18].

The aim to this study was to identify metabolic changes in maternal plasma of pregnant women, who delivered preterm after spontaneous onset of labor with no prior history or defined risk factors for preterm birth.

## 2. Material and Methods

### 2.1. Biological Sample Collection

The study protocol was approved by the Local Ethical Committee of Medical University of Bialystok, Poland (no ethics committee approval: R-I-002/392/2013). Informed written consents were obtained after detailing the study and protocols to each of the study participants.

Patients were recruited in 3 Polish tertiary centers: Department of Perinatology and Obstetrics, Medical University of Bialystok, Institute of Obstetric and Emergency Medicine, University of Rzeszow, and Department of Obstetrics and Pathology of Pregnancy, Medical University of Lublin. All patients were Slavic ethnicity. Plasma was collected directly after admission to the hospital, before steroid or tocolytic therapy.

Metabolomic analysis was performed on plasma collected from the following groups: group I—patients who delivered preterm between 24 and 37 weeks of pregnancy (*n* = 57), group Ia—patients subdivided from group I, who delivered preterm (between 24 and 37 weeks of pregnancy) within 7 days after diagnosis (*n* = 37), group II—patients with symptoms of threatening preterm labor between 23 and 37 weeks of pregnancy who gave birth at term (*n* = 49), and group III—patients who had blood collected during natural childbirth at term after normal pregnancy, between 38 and 41 weeks of pregnancy (*n* = 25).

The diagnosis of PTB was made according to regular uterine contractions resulting in cervical dilatation (*n* = 30) or preterm premature rupture of membranes (PPROM) (*n* = 27). PPROM was confirmed with Amnisure test (Qiagen).

Gestational age of each patient was determined based on first trimester ultrasound. All newborns were born alive, and there was no previous risk factors in patients for the occurrence of preterm delivery. Among neonates, there was no preterm or term small for gestational age neonate (SGA).

All patients were Slavic ethnicity.

Exclusion criteria were women with all indicated preterm deliveries such as the following: multiple gestation, pregnancy-induced hypertension, diabetes, kidney disease (creatinine concentration above 2 mg/dL in the blood), and other complications during pregnancy, such as thrombocytopenia, systemic disease, thrombophlebitis, steroids, and antibiotics within 72 hours prior to blood sampling, cervical incompetence and cervical cerclage, and finally clinical chorioamnionitis (at least one temperature elevation of >37.8°C, tachycardia, uterine tenderness greater than expected, white blood cell (WBC) count above 18,000, and unpleasant vaginal odor). Exclusion of chorioamnionitis was done to provide more homogeneity to our case group as our aim is to restrict this study to spontaneous labor group with no underlying etiology or confounding factors leading to clinical chorioamnionitis.

Each patient had 10 mL of peripheral blood collected. The blood was then centrifuged, and after that, the plasma was separated and frozen at −80°C temperature.

### 2.2. Sample Preparation

Plasma was stored at −80°C until the day of analysis. Samples were prepared as previously described with minor changes [19]. Briefly, protein precipitation and metabolite extraction were performed by adding 1 volume of plasma to 3 volumes of cold (−20°C) mixture of methanol and ethanol (1 : 1, *v:v*). Samples were then vortex-mixed for 1 min and left standing on ice for 5 min. The pellet was removed by centrifuging at 16100 ×g for 20 min at 4°C, and the supernatant was filtered through a 0.22 *μ*m nylon filter directly to chromatographic vial. Due to necessity of ion source cleaning, the whole set of samples was divided into 4 batches. Each batch was formed by combining close to 1/4 of randomly chosen representatives of each group. Quality control (QC) samples were prepared by pooling equal volumes of plasma from each of the 40 samples from the first analytical batch. QC samples were independently prepared from this pooled plasma for each of 4 batches following the same procedure as for the rest of samples. QC samples were analyzed at the beginning of the run and every 8 samples throughout the run to provide a measurement not only of the system stability and performance [20] but also of the reproducibility of the sample treatment procedure. The number of 8 patient samples was selected as an analytical run for inserting QC samples taking into account the total time of the whole experiment and authors' personal experience.

### 2.3. Plasma LC-MS Analysis

Samples were analyzed by an HPLC system (1260 Infinity series, Agilent Technologies, Waldbronn, Germany) consisting of a degasser, binary pump, and thermostated autosampler maintained at 4°C connected to an Agilent Technologies QTOF (6530) mass spectrometry detector. Electrospray ionization (ESI) was used as an ion source. Samples (10 *μ*L) were injected onto a reversed-phase column (Discovery HS C18 150 mm × 2.1 mm, 3 mm; Supelco) with a guard column thermostated at 40°C. The system was operated in positive and negative mode at flow rate 0.6 mL/min with solvent A—water with 0.1% formic acid—and solvent B—acetonitrile with 0.1% formic acid. The gradient started from 25% B to 95% B in 35 min and returned to starting conditions in 1 min, keeping the reequilibration until 45 min. The detector operated in full scan mode from 50 to 1000 m/z for positive mode and from 50 to 1100 m/z for negative mode with a scan rate of 1 scan per second. Accurate mass measurements were obtained by online mass correction to reference masses delivered continuously during analyses. Reference masses at m/z 121.0509 (protonated purine) and m/z 922.0098 (protonated hexakis (1H,1H,3H-tetrafluoropropoxy) phosphazine or HP-921) were used in positive ion mode, whereas m/z 112.9856 (TFA anion) and m/z 1033.9881 (hexakis (1H,1H,3H-tetrafluoropropoxy) phosphazine or HP-0921) were applied in negative ion mode. The capillary voltage was set to 3000 V for positive and 4000 V in negative ionization mode, and the nebulizer gas flow rate was 10.5 L/min. Randomized samples were analyzed in two separate runs (first for positive and second for negative mode) in four batches.

### 2.4. Data Analysis

The resulting data files were cleaned of background noise and unrelated ions by the Molecular Feature Extraction (MFE) tool in the Mass Hunter Qualitative Analysis B.05.00 Software (Agilent Technologies). The MFE algorithm group ions related by charge state, isotopic distribution, and/or the presence of adducts and dimers by using the accuracy of the mass measurements. The MFE then creates a list of all possible components as represented by the full TOF mass spectral data. Each compound is characterized by mass, retention time, and abundance. Parameters selected for data extraction by the MFE were similar to those described previously [21]. The background noise limit was set to 500 counts, and to find coeluting adducts of the same feature, the following adduct settings were applied: +H, +Na, and +K in positive ionization and –H and +HCOO for negative ionization. Neutral loss of water was also included. Due to retention time shifts during LC-MS analyses samples were multialigned using Mass Profiler Professional (B.12.1, Agilent Technologies). Then filtering step was applied to clean the data matrix from random signals and to remove metabolic features with excessive drift in signal, retention time, or accurate mass. Data were filtered by removing the features that were present in less than 50% of QC samples and with coefficient of variation above 30%. Further filtering aimed to choose the features that were present at least in 90% of representatives in any of studied groups. Subsequently, each metabolic feature in each subject was corrected by a change in signal for the same metabolic feature in “bracketing” QC samples within an analytical batch [20]. Further data were transformed by applying common logarithm to intensities in order to approximate a normal distribution (MS Excel (Microsoft)).

### 2.5. Statistical Analysis

Multivariate analyses for qualification of data and classification of studied groups were performed using partial least square discriminant analysis (PLS-DA) and orthogonal PLS-DA methods (Simca-P+ 12.0, Umetrics). Parameters *R*^2^ (explained variance) and *Q*^2^ (predicted variance) were calculated and used for the assessment of model's performance and predictive abilities. Multivariate models were validated by cross-validation and permutation tests. Cross-validation of the OPLSDA models was performed by using 7-fold cross-validation approach. The original set of samples was divided randomly into 7 subsets. Six subsets were used to build the model, and the last subset was predicted. The cross-validation procedure was repeated 7 times until all samples had been predicted at least once and only once. Based on cross-validation, the *Q*^2^ values were calculated giving the estimation of the predictive ability of the model. Additionally, the results were used to build the classification table and based on that, the percentage of correctly classified samples was calculated (CC (correctly classified)).

In PLS-DA models for three groups (group I, group II, and group III), QC samples were predicted to check the quality of data. OPLS-DA models were built for four comparisons of two selected groups (group I versus group II, group Ia versus group II, group I versus group III, and group II versus group III), and group classification was examined. Models were built for both polarities separately.

Further univariate statistical analysis was performed. Descriptive statistics including mean concentration and standard error of the mean concentration were calculated for selected metabolites, henceforth called features. Aiming at discovery of statistically significant differences between considered groups of patients in metabolites' concentrations, either fitting an analysis of variance model [22] was carried out or nonparametric method (Wilcoxon rank-sum test) was applied [23]. To address the issue of multiple comparisons, false discovery rate (FDR) *p* value correction procedure was used [24]. In order to determine which statistical method is appropriate to employ, the normality of the features' distribution and the homogeneity of variances were investigated. In situations, when at least one of the tests used to check the violation of assumptions mentioned above gave a statistically significant result, the nonparametric approach was adapted. The normality of features' distribution was checked with the Shapiro-Wilk test [25], whereas the homogeneity of variances with the Levene's test [26]. It must be mentioned that only pairwise comparisons were investigated (group I versus group Ia, group I versus group II, group Ia versus group II, group I versus group III, and group II versus group III), since these collations constituted the objective of the study. We also compared the patients in which preterm labor started with uterine contractions (*n* = 30) to patients with PPROM (*n* = 27). Features, which distribution statistically significantly differed in compared groups, underwent further investigation for two main reasons: (i) checking their classification accuracy, that is, discrimination capability (distinguishing studied groups) and (ii) validation, that is, confirmation of obtained results stating that a particular feature assures nonrandom classification. To address these issues, for each statistically significant feature, ROC curve analysis was performed, which involved (among others) the following: (i) determination of the optimal threshold values with the Youden method [27], (ii) construction of the 95% confidence intervals for areas under the ROC curves, and (iii) testing whether the area under the particular ROC curve (AUC) was significantly greater than 0.5 (random classification) with the DeLong method [28]—*p* values for the AUCs are reported. Computations concerning the ROC curve analysis were carried out with the functions provided by the pROC package [29]. In additional to the ROC curve analysis, the overall classification accuracy—taking into consideration all statistically significant features—was checked with the support vector machine (SVM) [30] classifier with the radial basis kernel and leave-one-out as a cross-validation technique. Missing values for individual metabolites were imputed using median in concordance with the group adhesion. Packages e1071 and kernlab enabled carrying out the examination of the classification capability of selected metabolites. The R software environment was used for all calculations. Significance level alpha set to 0.05 was employed for all evaluations.

### 2.6. Metabolite Identification

Identification of compounds that were found to be significantly changing between any two compared groups was performed by searching the publicly available databases: METLIN (http://www.metlin.scripps.edu/), HMDB (http://www.hmdb.ca/), and LIPID MAPS (http://www.lipidmaps.org/). In addition, the identity of compounds was confirmed by LC-MS/MS analysis by using a QTOF (model 6530, Agilent Technologies). Experiments were repeated with identical chromatographic conditions to the primary analysis. Ions were targeted for collision-induced dissociation (CID) fragmentation on the fly based on the previously determined accurate mass and retention time. Comparison of the structure of the proposed compound with the fragments obtained can confirm the identity. Accurate mass data and isotopic distributions for the precursor and product ions can be studied and compared to spectral data of reference compounds, if available, obtained under identical conditions for final confirmation.

## 3. Results

The clinical characteristic of patients is presented in Table 1. The parameters differentiating groups were, as predicted, gestational age at collecting samples and gestational age at birth in weeks.

After LC-MS analysis, 131 chromatograms of samples and 24 of QCs were aligned and cleaned from random signals. In total, 374 features in positive ionization mode (out of 73,649) and 375 features in negative ionization mode (out of 57,411) were selected for the further data treatment.

Subsequently, the univariate analysis was performed as described in Material and Methods giving 168 significantly changing features in four studied comparisons. Finally, identification of selected features was performed by searching against commercial databases and by LC-MS/MS analysis. It was possible to identify 51 metabolites in both polarity modes using three databases: METLIN, HMDB, and LIPID MAPS. Identified metabolites are summarized in Table 2 including the percentage of change between the compared groups and the coefficients of variation for the abundances of these compounds in QCs (CV). Percentage of change was calculated based on the difference of the average of signal's abundance of a selected metabolite between studied groups divided by the average of signal's abundance in the second group. In case when a metabolite was present and significant in both polarities (tryptophan, linoleic acid, docosapentaenoic acid, and glycocholic acid), the results are shown only for the one with lower CV (QC) and a higher statistical significance.

The discriminating metabolites were grouped according to their characteristics. Classes of perturbed metabolites included 3 amino acids, 11 free fatty acids, 9 lipids, 9 hormone metabolites, 8 bile acids, and 11 other metabolites (Table 2).

The greatest amount of statistically significant difference in metabolites was between group II and group III. This may be due to the fact that these groups differ in week of pregnancy, when the blood was collected and the fact of starting labor. Comparing these groups, we observed significant changes in amino acids, fatty acids, lipids, hormones, bile acids, and eight metabolites classified to the group of other metabolites.

When we compared group I versus group II, we noticed that significantly different metabolites belong mainly to fatty acids. Only one amino acid—tryptophan—and one hormone metabolite—pregnenolone sulfate—were significantly different between these two groups. More statistically significant differences in amino acids and fatty acid abundance were found when, from group I, we divided patients, who delivered up to seven days after the start of the preterm labor symptoms (group Ia) and compared them with the group of threatened preterm labor (group II).

Comparing group I with group III, no significant changes were found in amino acids and fatty acids. The mostly differentiating metabolites between these two groups were hormones and their conjugates (decreased in group I) and bile acids (increased in group I).

We did not find any statistically significant differences between groups I (*n* = 57) and Ia (*n* = 37) and between the groups of patients with preterm uterine contractions (*n* = 30) and PPROM (*n* = 27).

**Figure 1:**
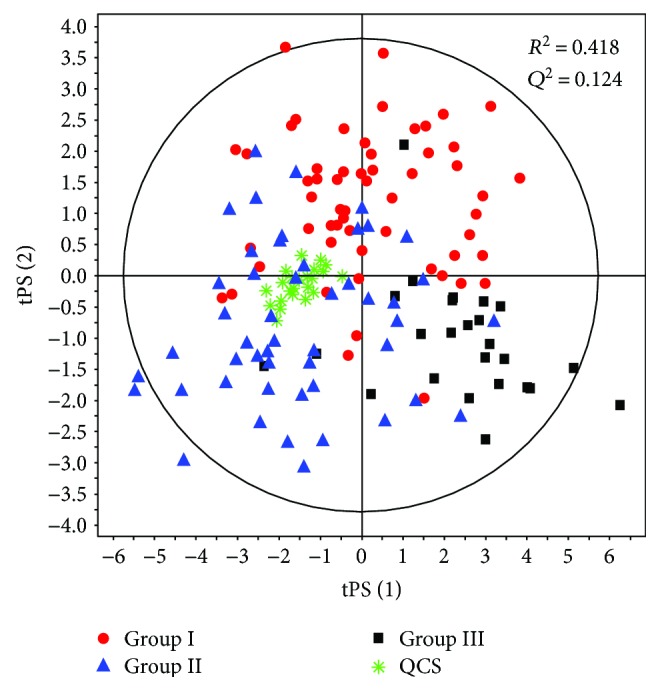
PLSDA score plot for groups I, II, and III with QC predicted (obtained for 374 selected variables from positive polarity mode). *R*^2^ and *Q*^2^ parameters are given for the two first components. Investigated groups are marked as follows: group I—red dots; group II—blue triangles; group III—black squares; and QCs—green stars.

For quality checking of analytical procedure, PLS-DA models for three groups of patients were built for both polarity modes (ESI+ and ESI−) and QC samples were predicted in the plots (Figure 1). Classification of QC samples and their clustering in the plots indicated correctness of the data (Figure 1). QC samples were not in the center of the plot; they were slightly moved to the left side of the plot characteristic for group I and II samples. The plasma for QCs was prepared from polled plasma from samples from first analytical batch containing 18 samples from group I, 16 from group II, and 6 from group III. Therefore, QC profiles were more similar to group I and II profiles.

**Figure 2:**
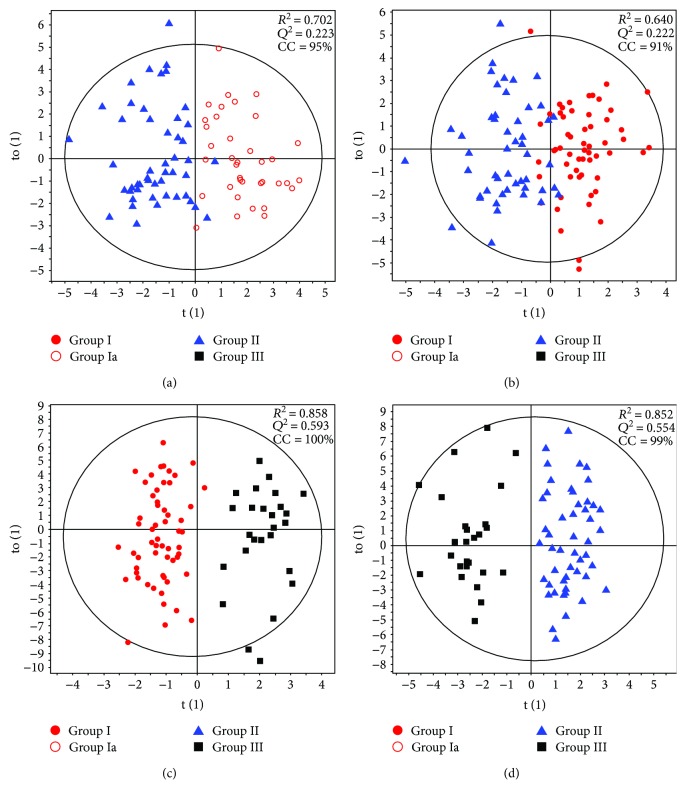
OPLSDA models compared with the studied groups (obtained for 374 selected variables from positive polarity mode). (a) OPLSDA for group Ia versus group II, correctly classified. (b) OPLSDA for group I versus group II. (c) OPLSDA for group I versus group III. (d) OPLSDA for group II versus group III. *R*^2^ and *Q*^2^ parameters are given for the first component for each model. CC: percentage of correctly classified samples according to classification table. Investigated groups are marked as follows: group I—red dots; group Ia—red circles; group II—blue triangles; group III—black squares.

Multivariate PLS-DA models were built to check the classification of studied groups, and OPLS-DA models were used for better visualization of classification plots. The models showed a clear separation of groups in the studied comparisons: group Ia versus group II, group I versus group II, group I versus group III, and group II versus group III (Figures 2(a)–2(d)). The best models were obtained for comparing group I or group II versus group III, but the model of group I versus group II had a relatively low *R*^2^ and *Q*^2^ values. Nevertheless, all models positively underwent validation procedure. The models created from profiles from ESI+ ion mode had slightly better quality parameters than those for ESI− (data not shown). The models strongly suggested that there was a clear pattern of discriminant metabolites between the studied groups. Based on classification table generated for OPLS-DA models of plasma fingerprints, we can observe that observations are classified with high probability to the correct class (correctly classified: CC values on score plot in Figure 2).

For significant metabolites, we also calculated SVM classification accuracy. The results are given in Table 3. The best classification accuracy was achieved between group I and group III (88 and 90% for ESI+ and ESI−, resp.). The classification accuracy between group II and group III was also high (88 and 85% for ESI+ and ESI−, resp.). We were able to classify group Ia and group II with overall accuracy (84% and 83% for ESI+ and ESI−, resp.). We found the lowest classification accuracy comparing group I versus group II (78 and 69% for ESI+ and ESI−, resp.). It is notable that measuring only 4 metabolites (palmitoleic acid, linolenic acid, and two unknowns of monoisotopic masses 586.4934 and 643.4026) in ESI− could classify group I and group II with 69% of accuracy.

Despite the fact that using univariate statistical analysis, the biggest amount of significant differences was between group II and group III, the best classification accuracy was achieved between group I and group III.

## 4. Discussion

We found that the most significant metabolites differentiating the studied groups belonged to the fatty acids. Saturated fatty acids (lauric acid, myristic acid, and hydroxymyristic acid) and also unsaturated fatty acids: omega 9 (palmotoleic acid and octadecenoic acid), omega 6 (linoleic acid, arachidonic acid, and eicosadienoic acid), and omega 3 (linolenic acid, docosahexaenoic acid, and docosapentaenoic acid) were different between the studied groups. Interestingly, both anti-inflammatory omega 3 [31] and proinflammatory omega 6 fatty acids [32] have the same character of change—decreased in group II comparing with groups I, Ia, and III and no significant change between groups I and III. The supplementation of omega 3 fatty acids, especially docosahexaenoic acid (DHA), is reported to have beneficial effect during pregnancy [33]. However, in our study, higher concentration of DHA was seen in patients delivered preterm (groups I and Ia) when compared with threatened preterm delivery (group II), but there was no significant change between preterm and term birth groups. Arachidonic acid, one of the omega 6 fatty acids, is a precursor of group II prostaglandins (PGE-2 and PGF-2*α*) that cause uterine contractile activity and may play an important role in the initiation of labor [34]. Too much arachidonic acid can induce uterine contractions and start of the delivery. In our study, this fatty acid also did not change significantly between the groups of preterm and term births.

It is difficult to determine if the high level of fatty acids in term pregnancies is associated with gestational age or is a consequence of initiation of term parturition. In the study performed by Lindsay et al. with the use of LC-MS/MS analysis, there was no statistically significant difference in the fatty acid concentration in plasma between the second and third trimesters of pregnancy [35]. Our results demonstrate that the presence of measurable concentration of fatty acids may be an indicator of labor regardless it is term or preterm.

The concentration of metabolites from the lipid group lysophosphatidylcholines (Lyso-PC (LPC)) and lysophosphatidylethanolamines (Lyso-PE (LPE)) decreased in groups I, Ia, and III in comparison with group II, but significant differences were found almost only between groups II and III. Lysophospholipids are the result of the phospholipase A2 activity. Phospholipase A2 hydrolyses phospholipids, and fatty acids and lysophospholipids are released. However in our study, changes in lysophospholipids are opposite to the changes in the group of fatty acids, where the concentration of metabolites in group II were lower than that in other groups.

Amniotic fluid metabolome study of pregnant women with PTB with and without intra-amniotic infection (IAI) and women with preterm labor (PTL) who delivered at term found a decrease in carbohydrates in both PTB groups compared to the group of PTL who delivered at term [36]. We did not observe similar differences in our study, where no significant change in carbohydrate (D-fructose/D-glucose) concentration in plasma of patients from preterm birth groups I and Ia compared to false preterm birth group II was found, but their levels were significantly elevated in group II when compared to group III. Romero et al. study also revealed increase in amino acids in PTL with intra-amniotic infection, when compared with PTL without IAI and PTL term delivery groups [36]. We found a decrease in amino acids in plasma of patients, who delivered preterm (groups I and Ia) compared to women with threatened preterm labor (group II). Lower level of amino acids might be due to their use as a source of energy or may be connected with oxidative stress—one of the factors which can induce preterm birth [37].

Among the metabolites found in our study, two amino acids histidine and tryptophan may be related with oxidative stress. Both of these amino acids had similar character of change with the compared groups and were lower in groups I and Ia when comparing with group II. In previous studies, histidine was negatively correlated with oxidative stress in obese women [38], while tryptophan tested in human milk acted as an inducer of oxidative stress [39]. What is surprising in our study is tryptophan decrease in preterm birth group had the highest significance and this amino acid was significantly higher in group II comparing with group III.

The concentration of 9 hormones and their metabolites was significantly higher in group III compared with group I and group II. When we compared group I with group II, only one metabolite pregnenolone sulfate, decreasing in group I, was significantly different. Progesterone is a steroid hormone produced initially in the corpus luteum and then in the placenta. Analogs of this hormone are widely used in the prevention of miscarriage and premature birth, although its mechanism of action is not entirely clear [40]. In our study, samples from group I were collected at about 30 weeks of pregnancy, from group II at approximately 32 weeks of pregnancy, and from group III at about 39 weeks of gestation. The higher concentration of hormone metabolites in group III may be due to the fact that the concentration of hormones depends on the week of pregnancy, not on the start of the labor. Similar results were presented in the work of Stamatelou et al. [41]. They demonstrated lower concentration of progesterone in the active phase of labor in patients giving birth prematurely when compared with patients giving birth on time. In this study, it was also shown that progesterone levels measured in patients from 28 to 34 weeks of gestation were lower in PTB patient group than in TD patient group. In another study conducted by López Bernal et al., they investigated differences in the production of progesterone, cortisol, and prostaglandin E in chorion and decidua cells collected from four groups of patients, following spontaneous labor at term, elective cesarean section at term, induced labor at term, and uncomplicated PTB [42]. In contrast to our study, where differences in the concentration of progesterone and cortisol levels between the groups were found in plasma, the above work showed no such differences among groups.

Metabolites, which correspond to bile acids, were significantly higher in groups I and II when compared with group III. In the metabolomic study performed by Menon et al., where amniotic fluid samples were searched, the higher concentration of bile acids was found in preterm labor and higher gestational age samples [17]. This is in contrast to our study where the concentration of bile acids was the highest in group II. In recent papers, the level of bile acids was measured mainly in the cholestasis. The cholestasis is the pathology of pregnancy, which occurs due to hypersensitivity to the hormones normally produced during pregnancy, and it increases the incidences of preterm birth and stillbirth [43].

Eleven metabolites from our study were not classified to any group. Most of them were not described in the previous studies about PTB.

In group Ia, higher level of anandamide was found when compared with group II. Anandamide is an organic chemical compound from the group of psychoactive cannabinoids found in living organisms. It was derived from arachidonic acid metabolism. In the study performed by Mitchell et al., amnion and choriodecidua tissues collected during term cesarean section, before the onset of labor, were stimulated by anandamide. It caused enhanced PGE2 production in amnion and chorion after stimulation [44]. These results combined with our results suggest the role of cannabinoids, such as anandamide in the start of labor.

Recent metabolomic preterm birth studies, which were performed on different biological samples, indicated changes in patients' metabolism; however, it was usually referred to other metabolites than those found in our study [45–47]. All metabolomics give the basics for targeted research to identify specific substances. What is interesting is one of the researches using cervicovaginal fluid collected at 20 weeks of gestation for metabolomic study with the use of gas chromatography-mass spectrometry did not indicate changes in preterm birth [48]. It would be very valuable to perform a similar study in plasma or urine; the collection of which is noninvasive as well as the cervicovaginal fluid collection.

We searched all the patients, which took part in the study for the presence or absence of infection and clinical chorioamnionitis. Patients with clinical or laboratory signs of infection were excluded from the study. The limitation of our research is the lack of histopathological examination of the placenta, which could reveal latent infection.

The other weakness of the study was the lack of data about neonates after leaving the hospital. Therefore, we do not know whether other potential neonatal disease would not have affected the metabolic profile of patients with preterm birth in our study.

## 5. Conclusion

Our study revealed the changes in individual metabolites and metabolomic profiles between the studied groups. The future studies between patients in different weeks of pregnancy before the pathologies start to develop should be performed. It will demonstrate if the changes in metabolomic profiles depend on pathology of pregnancy or gestational age.

## Figures and Tables

**Table 1:** Clinical characteristic of the patients.

	Group I (*n* = 57)	Group Ia (*n* = 37)	Group II (*n* = 49)	Group III (*n* = 25)
Maternal age (mean ± SD)	28.28 ± 6.32	28.49 ± 6.483	28.82 ± 5.195	27.76 ± 3.972
Number of pregnancies (mean ± SD)	2.105 ± 1.484	2.054 ± 1.290	1.694 ± 0.962	1.640 ± 0.757
Gestational age at collecting of samples in weeks (mean ± SD)	30.09 ± 3.419	30.7 ± 3.534	31.73 ± 3.712	39.60 ± 1.155
Gestational age at birth in weeks (mean ± SD)	31.75 ± 3.361	30.89 ± 3.462	39.16 ± 1.419	39.68 ± 1.069
Newborn sex (female : male)	25 : 32	16 : 21	29 : 20	10 : 15
BMI at the beginning of pregnancy (mean ± SD)	21.73 ± 3.61	21.80 ± 3.85	22.56 ± 4.55	21.84 ± 3.03
Present BMI (mean ± SD)	25.05 ± 4.52	25.26 ± 5.09	26.21 ± 4.26	27.63 ± 3.79

**Table 2:** List of identified metabolites significantly different between the studied groups.

Compound	Group I versus group II				Group Ia versus group II				Group I versus group III				Group II versus group III				
	Change (%)	*p* value	AUC	Greater *p* value	Change (%)	*p* value	AUC	Greater *p* value	Change (%)	*p* value	AUC	Greater *p* value	Change (%)	*p* value	AUC	Greater *p* value	CV (%)
*Amino acids*																	
Lysine^(*p,a*)^	−8	—	—	—	−13	—	—	—	8	—	—	—	18	^∗^	0.66	^∗∗^	7
Histidine^(*n,a*)^	−17	—	—	—	−22	^∗^	0.71	^∗∗∗^	−6	—	—	—	13	—	—	—	14
Tryptophan^(*p,a*)^	−12	^∗^	0.66	^∗∗^	−18	^∗∗^	0.73	^∗∗∗∗^	7	—	—	—	22	^∗^	0.73	^∗∗∗∗^	15
*Fatty acids*																	
Lauric^(*n,a*)^	19	—	—	—	23	^∗^	0.69	^∗∗∗^	−1	—	—	—	−17	^∗^	0.68	^∗∗^	14
Myristic^(*n,a*)^	31	—	—	—	32	^∗^	0.71	^∗∗∗^	−3	—	—	—	−26	^∗^	0.73	^∗∗∗∗^	22
Hydroxymyristic acid^(*n,b*)^	29	—	—	—	39	—	—	—	−6	—	—	—	−27	^∗^	0.73	^∗∗∗^	18
Palmotoleic acid^(*n,a*)^	66	^∗^	0.71	^∗∗∗∗^	79	^∗∗^	0.75	^∗∗∗∗^	−10	—	—	—	−46	^∗∗∗^	0.80	^∗∗∗∗^	9
Linolenic acid (C18:3w3)^(*n,a*)^	64	^∗^	0.69	^∗∗∗∗^	84	^∗∗^	0.77	^∗∗∗∗^	3	—	—	—	−37	^∗∗^	0.75	^∗∗∗∗^	10
Linoleic acid (C18:2w6)^(*p,a*)^	44	^∗^	0.69	^∗∗∗^	61	^∗∗^	0.76	^∗∗∗∗^	−3	—	—	—	−32	^∗∗^	0.76	^∗∗∗∗^	10
Oleic acid^(*p,a*)^	46	^∗^	0.71	^∗∗∗∗^	68	^∗∗∗^	0.80	^∗∗∗∗^	−2	—	—	—	−33	^∗∗^	0.78	^∗∗∗∗^	6
Arachidonic acid^(*n,a*)^	22	—	—	—	33	^∗^	0.70	^∗∗∗^	2	−	−	−	−16	^∗^	0.69	^∗∗∗^	17
Eicosadienoic (C20:2)^(*n,a*)^	52	—	—	—	61	^∗^	0.73	^∗∗∗^	16	—	—	—	−23	^∗^	0.72	^∗∗∗^	18
Docosahexaenoic acid (DHA)^(*n,a*)^	18	—	—	—	30	^∗^	0.71	^∗∗∗^	−5	—	—	—	−19	—	—	—	17
Docosapentaenoic acid (C22:5w3)^(*n,a*)^	30	—	—	—	46	^∗^	0.69	^∗∗∗^	−9	—	—	—	−30	^∗^	0.72	^∗∗∗∗^	14
*Lipids*																	
C16 sphingosine-1-phosphate^(*n,a*)^	−14	—	—	—	−13	—	—	—	−18	^∗^	0.70	^∗∗∗∗^	−5	—	—	—	7
Lyso PC (14:0)^(*p,a*)^	−7	—	—	—	−16	—	—	—	27	—	—	—	36	^∗^	0.70	^∗∗∗^	11
Lyso PC/PC (15:0)^(*n,a*)^	−8	—	—	—	−9	—	—	—	23	—	—	—	33	^∗^	0.69	^∗∗^	13
Lyso PE (18:1)^(*p,a*)^	−7	—	—	—	−8	—	—	—	14	—	—	—	23	^∗^	0.69	^∗∗^	12
Lyso PE (18:2)^(*p,a*)^	−3	—	—	—	−5	—	—	—	25	—	—	—	29	^∗^	0.71	^∗∗∗^	11
Lyso PC or PC (18:3)^(*p,a*)^	−21	—	—	—	−26	—	—	—	28	—	—	—	61	^∗^	0.71	^∗∗^	26
LysoPE(20:5)^(*p,a*)^	2	—	—	—	−1	—	—	—	76	^∗^	0.68	^∗∗^	72	^∗^	0.71	^∗∗∗^	28
PS(O-18:0/0:0)^(*p,a*)^	−11	—	—	—	−17	—	—	—	31	^∗^	0.70	^∗∗∗^	47	^∗∗∗^	0.81	^∗∗∗∗^	10
TG (triglyceride)^(*n,b*)^	29	—	—	—	28	^∗^	0.70	^∗∗∗^	−12	—	—	—	−31	^∗∗∗^	0.80	^∗∗∗∗^	22
*Hormones*																	
Progesterone^(*p,a*)^	−8	—	—	—	−11	—	—	—	−33	^∗∗∗∗^	0.83	^∗∗∗∗^	−27	^∗∗∗^	0.79	^∗∗∗∗^	6
Pregnenolone/bolasterone^(*p,b*)^	−5	—	—	—	0	—	—	—	−49	^∗∗∗^	0.85	^∗∗∗∗^	−46	^∗∗∗^	0.84	^∗∗∗^	8
Pregnenolone sulfate^(*p,b*)^	−33	^∗^	0.71	^∗∗∗∗^	−45	^∗∗^	0.76	^∗∗∗∗^	−33	^∗^	0.75	^∗∗∗∗^	0	—	—	—	20
17-Hydroxypregnenolone sulfate^(*n,b*)^	−5	—	—	—	−10	—	—	—	−32	^∗∗^	0.76	^∗∗∗∗^	−28	^∗^	0.74	^∗∗∗^	12
11-Beta-hydroxyandrosterone-3-glucuronide^(*n,b*)^	−46	—	—	—	−42				−50	^∗∗^	0.83	^∗∗∗∗^	−7	^∗^	0.73	^∗∗∗^	29
Cortisone/aldosterone/prednisolone^(*p,b*)^	0	—	—	—	6	—	—	—	−27	^∗∗^	0.80	^∗∗∗∗^	−27	^∗∗∗^	0.83	^∗∗∗∗^	8
Cortisol/hydroxycorticosterone^(*p,b*)^	10	—	—	—	20	—	—	—	−20	—	—	—	−27	^∗^	0.73	^∗∗∗^	5
3b,16a-Dihydroxyandrostenone sulfate^(*n,a*)^	17	—	—	—	26	—	—	—	−30	—	—	—	−40	^∗^	0.71	^∗∗^	21
Testosterone meabolite^(*n,b*)^	−1	—	—	—	−1	—	—	—	−30	—	—	—	−30	^∗^	0.68	^∗∗^	15
*Bile acids*																	
Deoxycholic acid^(*n,a*)^	−36	—	—	—	−47	—	—	—	62	—	—	—	154	^∗^	0.72	^∗∗∗^	30
Glycochenodeoxycholate^(*n,a*)^	−12	—	—	—	−17	—	—	—	145	^∗∗∗^	0.81	^∗∗∗∗^	180	^∗∗∗∗^	0.87	^∗∗∗∗^	6
Chenodeoxyglycocholate/glycoursodeoxycholate/glycodeoxycholate^(*p,b*)^	−40	—	—	—	−51	^∗^	0.69	^∗∗∗^	61	—	—	—	166	^∗∗∗^	0.80	^∗∗∗∗^	10
Chenodeoxyglycocholate/glycoursodeoxycholate/glycodeoxycholate^(*n,b*)^	−17	—	—	—	−25	—	—	—	210	^∗^	0.78	^∗∗∗∗^	275	^∗∗∗^	0.83	^∗∗∗∗^	11
Chenodeoxyglycocholate/glycoursodeoxycholate/glycodeoxycholate^(*n,b*)^	−40	—	—	—	−51	—	—	—	25	^∗^	0.73	^∗∗∗^	109	^∗∗^	0.79	^∗∗∗∗^	6
Glycocholic acid^(*p,a*)^	−11	—	—	—	−12	—	—	—	92	—	—	—	116	^∗^	0.74	^∗∗∗^	15
TUDCA/TUDCA isomer/taurodeoxycholic acid^(*n,b*)^	−19	—	—	—	−32	—	—	—	74	^∗^	0.70	^∗∗^	116	^∗∗^	0.76	^∗∗∗∗^	7
TUDCA/TUDCA isomer/taurodeoxycholic acid^(*n,b*)^	0	—	—	—	−4	—	—	—	99	^∗^	0.68	^∗∗^	100	^∗^	0.68	^∗∗^	7
*Other metabolites*																	
Malonaldehyde^(*n,a*)^	−2	—	—	—	10	—	—	—	11	—	—	—	14	^∗^	0.71	^∗∗∗^	25
2-Hydroksybutyric acid/3-hydroksybutyric acid^(*n,b*)^	58	—	—	—	89	^∗^	0.71	^∗∗∗∗^	22	—	—	—	−23	^∗^	0.69	^∗∗^	29
Malic acid^(*n,a*)^	3	—	—	—	4	—	—	—	−20	^∗∗^	0.75	^∗∗∗∗^	−23	^∗∗^	0.79	^∗∗∗∗^	7
L-2-Amino-3-(1-pyrazolyl)propanoic acid^(*p,b*)^	−10	^∗^	0.68	^∗∗∗^	−13	^∗^	0.72	^∗∗∗∗^	−4	—	—	—	7	—	—	—	4
Carnitine^(*p,a*)^	−19	—	—	—	−23	—	—	—	21	—	—	—	49	^∗^	0.70	^∗∗∗^	28
D-Fructose/D-glucose^(*n,a*)^	−2	—	—	—	−4	—	—	—	9	—	—	—	12	^∗^	0.70	^∗∗∗^	7
N,N′-Dicyclohexyl-urea^(*p,a*)^	26	—	—	—	23	—	—	—	−51	^∗∗∗∗^	0.87	^∗∗∗∗^	−61	^∗∗∗∗^	0.91	^∗∗∗∗^	8
Sphingolipid: obscuraminol A/crucigasterin 277^(*p,b*)^	25	—	—	—	9	—	—	—	−26	—	—	—	−41	^∗^	0.76	^∗∗∗∗^	16
(4E,8E,10E-d18:3)Sphingosine^(*p,b*)^	49	^∗^	0.69	^∗∗∗^	64	^∗∗^	0.76	^∗∗∗∗^	12	—	—	—	−25	—	—	—	10
Anandamide (20:l, n-9)^(*p,b*)^	47	—	—	—	59	^∗^	0.71	^∗∗∗^	15	—	—	—	−22	—	—	—	23
Biliverdin^(*p,a*)^	1	—	—	—	27	—	—	—	−26	^∗^	0.71	^∗∗∗^	−27	^∗^	0.73	^∗∗∗∗^	9

QC CV: coefficient of variation of a metabolite calculated for QC samples; AUC: area under the curve from ROC validation; greater *p* value: corrected *p* value for AUC; ^∗^*p* value ≤ 0.05; ^∗∗^*p* value ≤ 0.01; ^∗∗∗^*p* value ≤ 0.001; ^∗∗∗∗^*p* value ≤ 0.0001; ^*n*^metabolites found in negative polarity mode; ^*p*^metabolites found in positive polarity mode; ^*a*^metabolites identified by MS/MS spectra and MS fragmentation pattern; ^*b*^metabolites identified putatively by exact mass data and isotopic pattern distribution.

**Table 3:** Number of significant features (total and identified) and SVM classification accuracy-obtained with the significant (after applying *p* value adjustment procedure) as predictors verified with the leave-one-out cross-validation technique.

	Number of significant features				Quality of classification	
	ESI+ total	ESI+ identified	ESI− total	ESI− identified	SVM ESI+	SVM ESI−
Group I versus group II	22	6	4	2	78%	69%
Group Ia versus group II	32	8	26	11	84%	83%
Group I versus group III	22	8	57	9	88%	90%
Group II versus group III	66	22	71	24	88%	85%
All	74	26	94	29	—	—
